# Delirium – are we doing enough prevention and basic management in acute settings?

**DOI:** 10.1192/bjo.2021.328

**Published:** 2021-06-18

**Authors:** Anna Watkins, Remy Flechais, Shah Tarfarosh

**Affiliations:** The John Radcliffe Hospital, Oxford University Hospitals NHS Foundation Trust

## Abstract

**Aims:**

To identify the prevalence of delirium and/or dementia on complex medicine wards.

To assess the use non-pharmacological prevention and management options in these patients.

**Background:**

Delirium, a common hospital syndrome, is often multi-factorial. So, the management needs not only treating a reversible cause but also minimising the factors that could increase the risk of developing delirium, or worsen its course.

The Scottish-Intercollegiate-Guidelines-Network (SIGN) and National-Institute-for-Health-and-Care-Excellence (NICE) guidelines outline non-pharmacological factors to reduce the risk of developing delirium, and for its management once established.

These factors include orientation, ensuring patients have their glasses and hearing aids, promoting sleep hygiene, maintaining optimal hydration and nutrition, early mobilisation, appropriate lighting and providing cognitively stimulating activities.

**Method:**

SIGN, NICE and local guidelines were used to develop a checklist of core non-pharmacological factors that minimise the risk of developing delirium and help in its management. Adherence to recommendations from these guidelines was thus evaluated in 4 Complex Medical Units of The John-Radcliffe Hospital (Oxford University Hospitals NHS Foundation Trust), cross sectionally. The data were collected by interviewing nursing staff on the wards, assessing the ward environment, reviewing nursing charts and electronic patient records.

**Result:**

There were 57 patients aged >65 years across all four wards, with average percentages of delirium and dementia patients being 46% and 34%, respectively. Nurses were unsure about their patients having hearing or visual aids in 41% and 29%, respectively. On all four wards there was no clear signage, no digital clock, no calendar, and earplugs were not offered. Overall, the use of non-pharmacological recommendations was sub-optimal across a number of items. After a month, when the notes were reviewed, it was found that 18 out of those 57 patients had passed away (32%) and the average length of stay for delirium/dementia patients was way more than the other patients during that admission.

**Conclusion:**

We found high rates of delirium and dementia and a lack of consistent use of recommended non-pharmacological strategies for their management. Better adherence to these could help shorten length of stay and improve patient outcomes.

Recommendations for patients with/at risk of delirium:

- Bedside board for each patient with the name of the ward/hospital, picture of the named nurse.
- Ensuring visible clock/calendar.
- Non-pharmacological delirium management checklist to be added to the daily nursing notes.

Emphasis on visual/hearing aids and daily reorientation.

- Appropriate lighting in the bays.
- Offer earplugs if not sleeping at night.

